# IL-23 Induces Atopic Dermatitis-Like Inflammation Instead of Psoriasis-Like Inflammation in CCR2-Deficient Mice

**DOI:** 10.1371/journal.pone.0058196

**Published:** 2013-03-05

**Authors:** Shannon K. Bromley, Ryan P. Larson, Steven F. Ziegler, Andrew D. Luster

**Affiliations:** 1 Center for Immunology and Inflammatory Diseases, Division of Rheumatology, Allergy and Immunology, Massachusetts General Hospital, Harvard Medical School, Boston, Massachusetts, United States of America; 2 Department of Immunology, University of Washington School of Medicine, Seattle, Washington, United States of America; 3 Immunology Program, Benaroya Research Institute at Virginia Mason, Seattle, Washington, United States of America; Dana-Farber Cancer Institute, United States of America

## Abstract

Psoriasis is an immune-mediated chronic inflammatory skin disease, characterized by epidermal hyperplasia and infiltration of leukocytes into the dermis and epidermis. IL-23 is expressed in psoriatic skin, and IL-23 injected into the skin of mice produces IL-22-dependent dermal inflammation and acanthosis. The chemokine receptor CCR2 has been implicated in the pathogenesis of several inflammatory diseases, including psoriasis. CCR2-positive cells and the CCR2 ligand, CCL2 are abundant in psoriatic lesions. To examine the requirement of CCR2 in the development of IL-23-induced cutaneous inflammation, we injected the ears of wild-type (WT) and CCR2-deficient (CCR2^−/−^) mice with IL-23. CCR2^−/−^ mice had increased ear swelling and epidermal thickening, which was correlated with increased cutaneous IL-4 levels and increased numbers of eosinophils within the skin. In addition, TSLP, a cytokine known to promote and amplify T helper cell type 2 (Th2) immune responses, was also increased within the inflamed skin of CCR2^−/−^ mice. Our data suggest that increased levels of TSLP in CCR2^−/−^ mice may contribute to the propensity of these mice to develop increased Th2-type immune responses.

## Introduction

Activated T cells and the cytokines they produce are thought to drive the pathogenesis of psoriasis [1]. Cytokines secreted by CD4^+^ T cells stimulate keratinocytes to proliferate and recruit inflammatory cells into the skin, promoting epidermal hyperplasia and inflammation. Because CD4^+^ T cells producing the T helper cell type 1 (Th1) cytokine IFN-γ are present in large numbers within psoriatic plaques [2], Th1 cells have long been considered the principal mediators of disease development. More recently, a role for Th17 cells in psoriasis has been recognized. Th17 cytokines, including IL-17A, IL-17F, and IL-22, are found at higher levels in psoriatic skin lesions than in non-psoriatic and normal skin [3], [4]. Additionally, IL-23, a Th17 growth and differentiation factor and its receptor are increased in psoriatic lesions [4], [5], [6]. Moreover, injection of wild-type (WT) mice with IL-23 reproduces several aspects of disease, including epidermal acanthosis, hyperkeratosis and a mixed dermal inflammatory infiltrate that includes mononuclear cells and granulocytes–the majority of which are neutrophils [7], [8], [9]. Finally, recent clinical data demonstrate a critical role for Th17 cytokines. Immunotherapies using antibodies targeting IL-17 [10], [11], [12] or IL-12/IL-23 [13], [14], [15], [16] are effective psoriasis treatments.

Several data suggest that chemokines and their receptors regulate the pathogenesis of inflammatory diseases, including psoriasis by regulating the recruitment of leukocytes into affected tissues. Th17 cells express the chemokine receptor, CCR6 [8], [17], [18], [19], [20], and recent studies demonstrate that the CCR6 ligand, CCL20 is up-regulated in psoriatic plaques [18], [21]. The finding that CCR6-deficient mice fail to develop psoriasiform pathology following intradermal injection with IL-23 supports a critical role for CCR6 in this inflammatory skin disorder [9]. The expression of many other chemokines within psoriatic lesions suggests that additional chemokine-driven mechanisms may also regulate disease development.

CCR2 has been implicated in the pathogenesis of several inflammatory diseases, and CCR2 antagonists have been developed. CCR2 is expressed on activated T cells–including Th17 cells [22], [23], as well as monocytes, macrophages, immature dendritic cells, γδ T cells and NK cells [24]. CCR2 binds multiple murine chemokine ligands: CCL2 (MCP-1), CCL7 (MCP-3) and CCL12 (MCP-5) [25]. CCL2 is expressed at high levels in psoriatic plaques by keratinocytes [26], [27], suggesting a potential role for CCR2 in psoriasis pathogenesis. A requirement for CCR2 in the development of Th17-mediated autoimmune inflammation has been demonstrated [28], [29]; EAE disease pathology in CCR2-deficient (CCR2^−/−^) mice is ameliorated. Protection from EAE is associated with a decreased IFN-γ response [28], although IL-17 and IL-22 cytokine production was not measured in these studies. In contrast, in a mouse model of collagen-induced arthritis, disease severity was exacerbated in CCR2^−/−^ mice, and this correlated with an increased Th17 response [30]. Thus, depending on the disease model, CCR2-deficiency may have an inflammatory or anti-inflammatory effect.

Recent studies have demonstrated that skewing CD4^+^ T cell phenotype within psoriatic plaques to a Th2-type immune response can ameliorate disease [31], [32], [33]. Treatment of psoriasis patients with subcutaneous injections of IL-4 polarizes lesional T cell responses to a Th2-type and decreases psoriasis severity [31]. Similarly, transdermal delivery of IL-4 expression plasmid ameliorates disease in a mouse model of psoriasis [32], [33]. Thus, induction of a Th2 phenotype of skin infiltrating lymphocytes correlates with disease improvement. In several models of inflammation, CCR2 blockade blunts Th1-type immune responses and enhances Th2-type immune responses. Studies using mouse models of infection [34], inflammation [28], [35], and graft rejection [36] have demonstrated that CCR2-deficient (CCR2^−/−^) mice exhibit enhanced Th2-type responses with increased production of IL-4 and IL-5, but decreased production of IFN-γ. Given this bias toward Th2 immunity in the absence of CCR2 signaling, and a potential role for CCR2 in the pathogenesis of psoriasis, we sought to examine whether CCR2-deficient mice are resistant to the development of IL-23-induced psoriasis.

To test the hypothesis that CCR2 is required for the development of psoriasis, we injected WT and CCR2^−/−^ mice intradermally with IL-23. We found that the skin of CCR2^−/−^ mice actually becomes more inflamed than that of WT mice, with increased ear swelling and epidermal thickening. Further, instead of psoriasis-like inflammation seen in WT mice, IL-23-induced cutaneous inflammation in CCR2^−/−^ mice resembled atopic dermatitis. This inflammation was associated with an increased Th2-type immune response. Although comparable numbers of IL-4-secreting CD4^+^ T cells were present in the draining lymph node and inflamed skin of WT and CCR2^−/−^ mice, increased numbers of eosinophils, mast cells and increased expression of IL-4 and TSLP were detected within IL-23 injected CCR2^−/−^ skin.

## Materials and Methods

### Mice

CCR2^−/−^ mice [37] on a C57Bl/6 background were bred at Massachusetts General Hospital and housed in a specific pathogen-free microisolator environment. C57Bl/6 mice were obtained from the Jackson Laboratory. All experiments were performed according to protocols approved by the Massachusetts General Hospital Subcommittee on Research Animal Care (OLAW Number: A3596-01).

### Intradermal IL-23 Injections and Ear Swelling Measurement

Cutaneous inflammation was induced by injecting the ears of anesthetized mice every other day with 20 µL PBS alone or containing 500 ng IL-23 (R&D Systems) using a 30-gauge needle attached to a 50 µL Hamilton syringe every other day for ten days. Ear swelling was measured each day immediately before injection, starting on day 0. Ear measurements were made using a pocket thickness gauge (Mitutoyo USA, Aurora, IL).

### Histology, Measurement of Epidermal Thickness and Eosinophil, Neutrophil and Mast Cell Quantitation

For histological assessment, ears were isolated and placed in 10% formalin. Formalin preserved ear skin was embedded in paraffin and sections were stained with H&E. Images were acquired and an investigator blinded to the genotype of the animals determined the percent of eosinophils or neutrophils among total leukocytes. 5 µm sections were stained with toluidine blue for mast cell quantitation. Epidermal thickness was evaluated using NIS-Elements software, and was calculated as: the area of the epidermis/the length of the epidermis.

### Fluorescence Immunohistology

Fluorescence immunohistology was performed on 10 µm frozen ear skin sections. The sections were air dried for 10 minutes, fixed in 4% paraformaldehyde for 8 minutes, and blocked with PBS containing 5% FBS and 2 µg anti-CD16/CD32 (Biolegend) for 30 minutes at room temperature. Sections were then washed twice in PBS 0.1% Tween and stained overnight with 1 µg of an affinity purified polyclonal antibody to mouse TSLP (R & D Systems, BAF555) diluted in PBS 0.1% saponin at 4**°**C. After washing with PBS 0.1% Tween on ice, sections were stained with 1 µg of AlexaFluor 488-conjugated donkey anti-goat IgG (Invitrogen) for 1 hour at room temperature. After again washing with PBS 0.1% Tween, sections were then stained with 1 µg/mL Hoescht in PBS for 5 minutes. Finally, sections were washed in PBS, mounted with ProLong Gold Antifade Reagent (Invitrogen), and examined under a fluorescence microscope.

### Processing of Ear Skin and Lymph Nodes for *ex vivo* Stimulation and Flow Cytometry

Injected ears were isolated and minced. Ear skin was then digested for 1 hour at 37**°**C in 5 mL HBSS containing 1 mg/mL collagenase D (Sigma). Cells were then filtered through 70 µm nylon mesh and washed in complete RPMI before stimulation. Lymph nodes were disrupted using a 1 mL syringe plunger in complete RPMI. Recovered leukocytes were passed through wire mesh to obtain single cell suspensions. Samples were counted using a hemacytometer.

### Intracellular Staining

Intracellular cytokine staining for IL-4 was performed after *in vitro* activation with PMA (50 ng/mL) and ionomycin (500 ng/mL) for five hours at 37**°**C. GolgiPlug (BD Biosciences) was added for the last four hours of stimulation. After stimulation, cells were incubated with Fc block (Biolegend) and stained with antibodies directed against CD3 and CD4. Cells were then fixed and permeabilized using Fix and Perm cell fixation and permeabilization kit (Invitrogen) and stained with antibody against IL-4.

### RNA Isolation and qPCR

Total RNA was isolated from mouse skin, and quantitative PCR was performed as described [38] with the Mx4000 Multiplex Quantitative PCR System (Stratagene). Primer sequences used for qPCR of β2m and CCL7 [39]; IL-4, IL-5, IL-13, IFN-γ and IL-25 [40]; IL-17A, IL-17F, and IL-22 [41] have been published. The following additional primer pairs were used: CCL2 F-TGG CTC AGC CAG ATG CAG T and R-TTG GGA TCA TCT TGC TGG TG; CCL12 F-GCT GGA CCA GAT GCG GTG and R-CCG GAC GTG AAT CTT CTG CT; TSLP F-ACG GAT GGG GCT AAC TTA CAA and R-AGT CCT CGA TTT GCT CGA ACT.

### TSLP Tissue Lysate ELISA

Ear skin was transferred into T-PER tissue protein extraction reagent (Thermo Scientific) containing a protease inhibitor cocktail (Roche) and homogenized using a Polytron (Kinematica:AG). Protein concentration was quantified using a BCA protein quantification assay. ELISA for TSLP protein was performed according to the manufacturer’s instructions (R&D Systems).

### Statistics

Comparisons were analyzed for statistical significance by Student’s t-test with Microsoft Excel software, with P<0.05 being considered significant.

## Results

### IL-23-induced Cutaneous Inflammation is More Severe in CCR2^−/−^ Mice than in WT Mice

**Figure 1:**
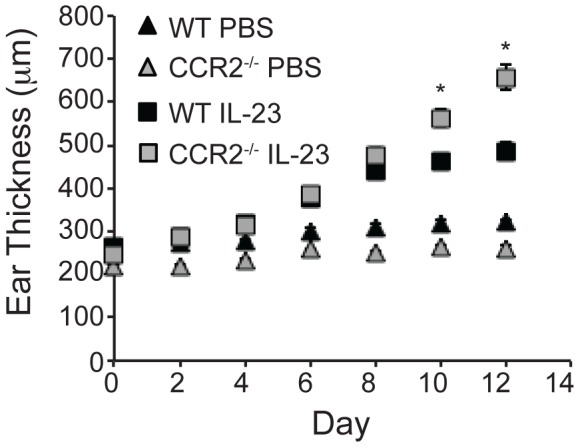
IL-23 injection induces increased inflammation in CCR2^−/−^ mice compared to WT mice. Ears of CCR2^−/−^ and WT mice were injected every other day with 20 µL PBS alone or containing 500 ng IL-23. Ear thickness was measured one day following each injection. Data are from at least 13 mice/group total in three separate experiments. *p<0.01 versus all other groups.

Expression of the CCR2 ligand, CCL2 by basal keratinocytes within psoriatic plaques has been detected [26], [27], suggesting a role for CCR2 in psoriasis pathogenesis. To determine the requirement of CCR2 for development of psoriasis, we examined whether CCR2^−/−^ mice are protected from the development of IL-23-induced psoriatic inflammation. We injected WT and CCR2^−/−^ mice intradermally in the ear with IL-23 every other day. As has been reported previously, WT mice develop severe ear swelling following intradermal IL-23 injection [7], [8], [9]. Ear thickness increased by more than 150 µm compared with PBS-injected WT control mice twelve days after initiation of IL-23 injections (Figure 1). Contrary to our hypothesis, CCR2^−/−^ mice actually developed more severe ear swelling than WT mice. Average ear thickness of CCR2^−/−^ mice increased more than 300 µm on day 12 (Figure 1).

### CCR2^−/−^ Mice Exhibit Increased Epidermal Thickness and Tissue Eosinophilia

**Figure 2:**
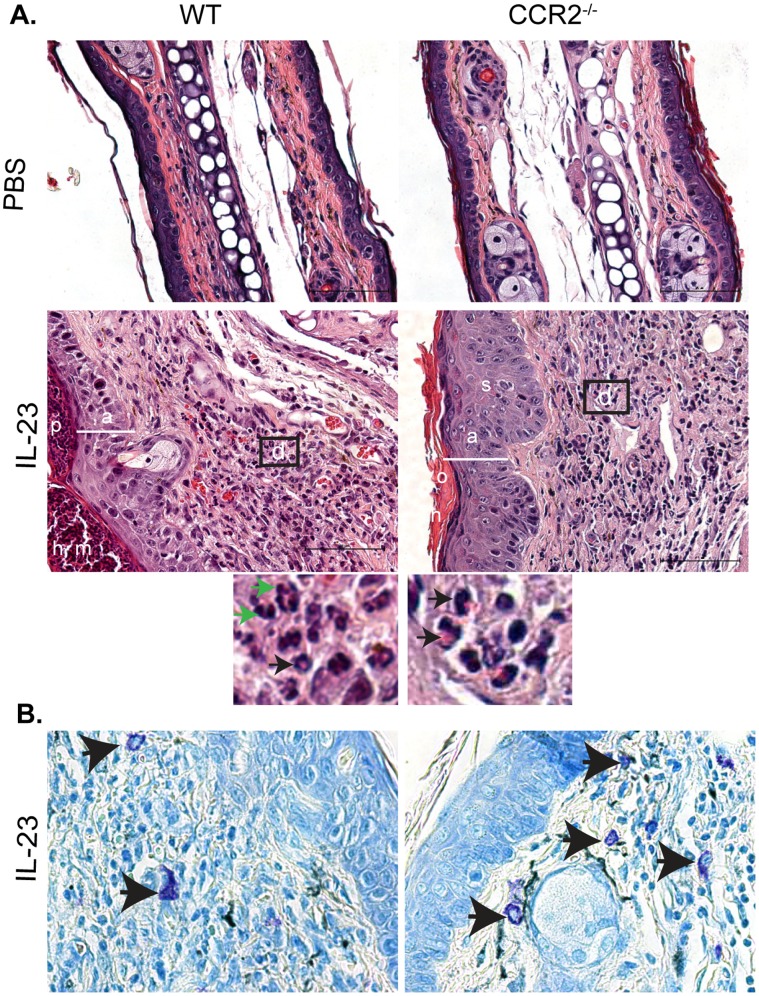
Eosinophils and mast cells accumulate in ears of IL-23-injected CCR2^−/−^ mice. (**A**) H&E-stained sections of ears from IL-23-injected WT and CCR2^−/−^ mice at day 12. a, acanthosis; h, hyperkeratosis; p, parakeratosis; o, orthokeratosis; d, dermal inflammatory infiltrate; s; spongiosis; m, intracorneal microabscess. Enlargements of the boxed areas within the WT IL-23 or CCR2^−/−^ IL-23 image are displayed below the corresponding photo. Black arrows indicate eosinophils. Green arrows indicate neutrophils. (**B**) Toluidine blue-stained sections of ear from IL-23-injected WT and CCR2^−/−^ mice at day 12. Arrows indicate mast cells.

**Figure 3:**
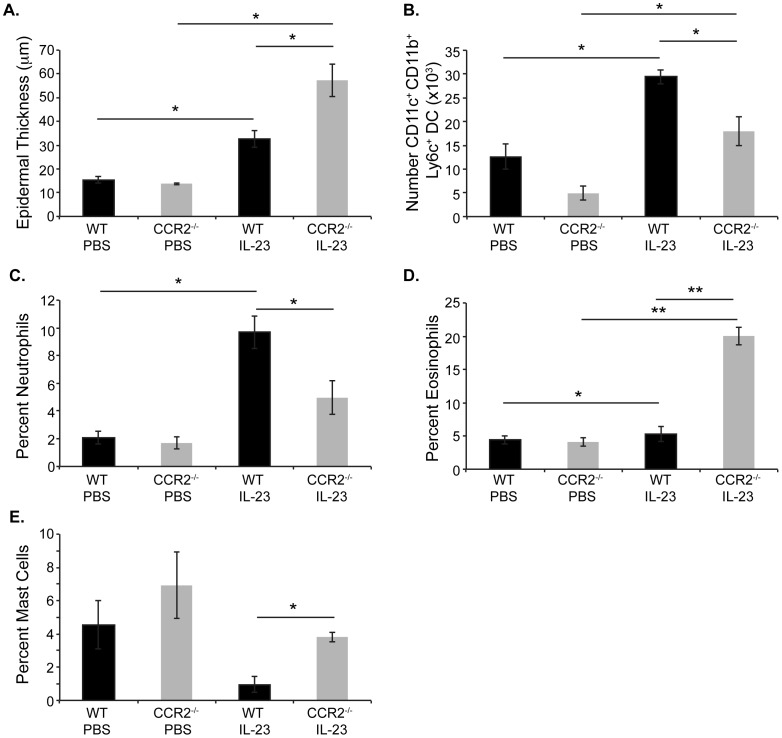
Increased epidermal thickness and accumulation of eosinophils and mast cells in ears of IL-23-injected CCR2^−/−^ compared to WT mice. (A) Average epidermal thickness was measured on H&E-stained sections. *p<0.02. Data are from two independent experiments with three-four mice/group. (B) Ears were digested with collagenase and recovered leukocytes were analyzed by flow cytometry to determine numbers of inflammatory dendritic cells (CD11c^+^ CD11b^+^ Ly6c^+^) within ears of IL-23 injected WT and CCR2^−/−^ mice. *p<0.05. Data are from one experiment with three mice/group. Average percent neutrophils (C) and eosinophils (D) among leukocytes in H&E sections of ears from IL-23-injected WT and CCR2^−/−^ mice at day 12. (E) Average percent mast cells among leukocytes in toluidine blue sections of ears from IL-23-injected WT and CCR2^−/−^ mice at day 12. Slides were imaged (x200, original magnification) and numbers of eosinophils or neutrophils among leukocytes were counted. Data are from four mice/genotype with at least three fields counted per mouse. *p<0.05; **p<0.005.

To further characterize the increased ear swelling observed in CCR2^−/−^ mouse ears, we examined H & E stained skin sections. WT mice injected intradermally with IL-23 exhibit several features of psoriasis, including epidermal acanthosis, hyperkeratosis and a mixed dermal inflammatory infiltrate that includes mononuclear cells and granulocytes– the majority of which are neutrophils [7], [8], [9]. Additionally, microabscesses within the cornified layer were readily observed in skin sections (Figure 2a). CCR2^−/−^ mice injected with IL-23 display many of these same characteristics – epidermal acanthosis, hyperkeratosis and a mixed dermal inflammatory infiltrate. However, several differences in the histology of IL-23-injected WT and CCR2^−/−^ skin were evident. First, CCR2^−/−^ mice developed increased epidermal thickening compared to WT mice (Figure 2a, 3a). Additionally, whereas WT mice developed parakeratosis, the stratum corneum of CCR2^−/−^ mice lacked nuclei (Figure 2a). Moreover, flow cytometric analysis demonstrated that although both IL-23-injected WT and CCR2^−/−^ skin displayed increased accumulation of inflammatory dendritic cells (CD11c^+^ CD11b^+^ Ly6c^+^) compared to PBS-injected control mice, more dendritic cells were identified in the ears of WT than CCR2^−/−^ mice (Figure 3b). Finally, while there was a mixed dermal inflammatory infiltrate in the ears of both WT mice and CCR2^−/−^ mice (Figure 2a), a greater percent of neutrophils were present in the inflamed skin of WT mice (Figure 3c). In contrast, an abundance of eosinophils (Figure 2a, 3d) and mast cells (Figure 2b, 3e) accumulated in the ears of CCR2^−/−^ mice suggesting the development of a Th2-type immune response resembling atopic dermatitis [42], [43].

### CCR2 Ligands are Expressed in WT and CCR2^−/−^ IL-23-injected Skin

**Figure 4:**
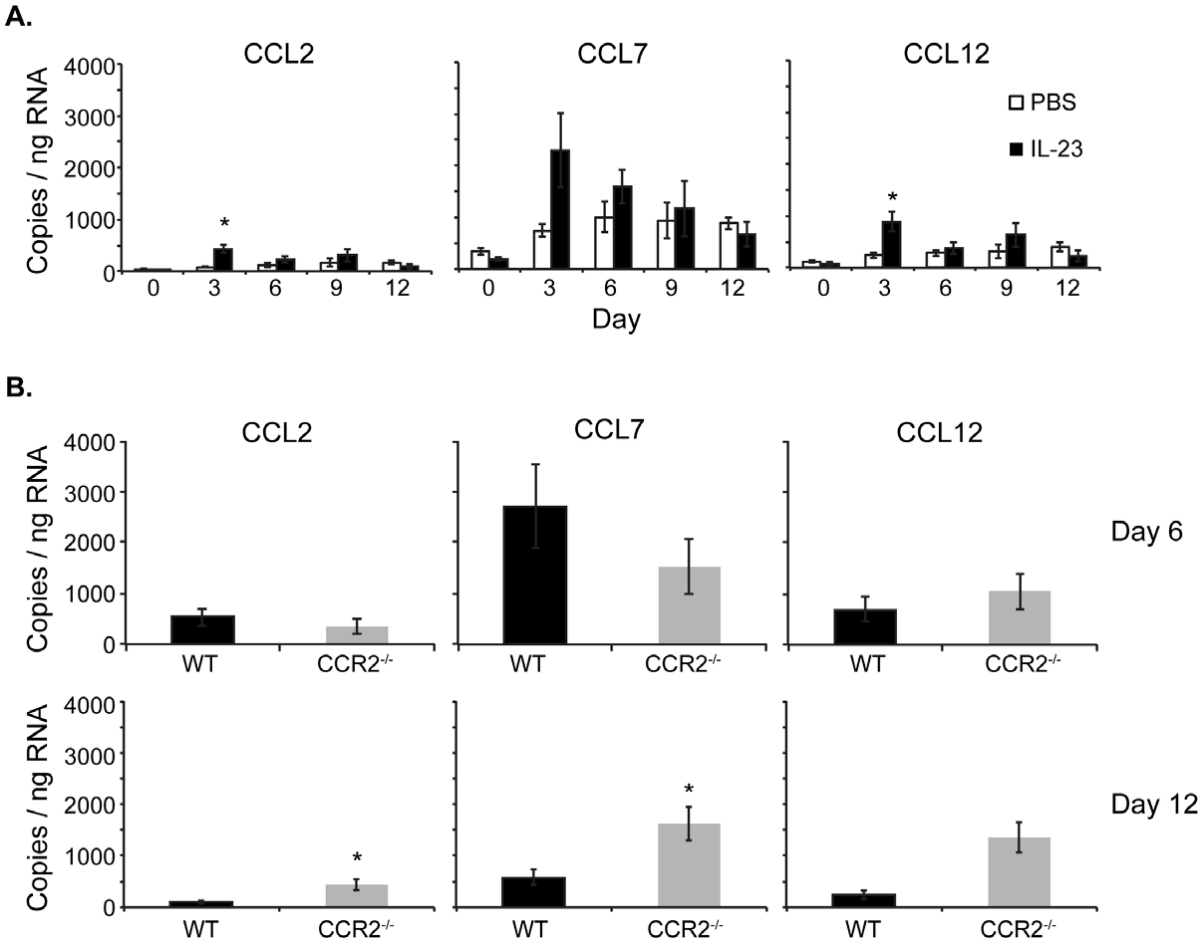
CCR2 ligands are expressed in IL-23-injected WT and CCR2^−/−^ mouse ears. (**A**) On days 0, 3, 6, 9 and 12, CCL2, CCL7 and CCL12 mRNA were measured by real-time RT-PCR from ears of PBS-injected or IL-23-injected WT mice. Average of three mice. (**B**) On day 6 and 12, CCL2, CCL7 and CCL12 mRNA was measured by real-time RT-PCR from ears of WT and CCR2^−/−^ IL-23-injected mice. Day 6 results are an average of 11 mice in 3 experiments. Day 12 results are an average of 8 mice in 2 experiments. *p<0.02.

The chemokine receptor CCR2 binds multiple murine ligands: CCL2, CCL7 and CCL12 [25]. Real time RT-PCR analysis of WT ears demonstrated that CCL2, CCL7 and CCL12 mRNA were most highly expressed on day 3–6 and then diminished by day 12 after initiation of IL-23 injections (Figure 4a). These results demonstrate that CCR2 ligands are expressed within the ears of IL-23-injected WT mice and could recruit CCR2^+^ cells. The CCR2 ligands were expressed at least as highly in the ears of IL-23-injected CCR2^−/−^ as WT mice on day 6 (Figure 4b). Thus, although CCR2^−/−^ cells cannot respond to CCR2 ligands, these results demonstrate that CCR2 is not required for CCR2 ligand production. At day 12, the CCR2 ligands remained elevated in CCR2^−/−^ ear skin compared to WT ear skin, perhaps as a compensatory mechanism for CCR2 deficiency or as a lack of CCR2 ligand scavenging in the absence of CCR2^+^ cells. Of note, CCL7 also signals through CCR3 [44], [45], a chemokine receptor that remains intact in CCR2^−/−^ mice and is expressed on basophils, mast cells and eosinophils [46], providing a potential mechanism for the increased recruitment of eosinophils into CCR2^−/−^ skin.

### IL-22 is Expressed in IL-23-injected WT and CCR2^−/−^ Ear Skin

**Figure 5:**
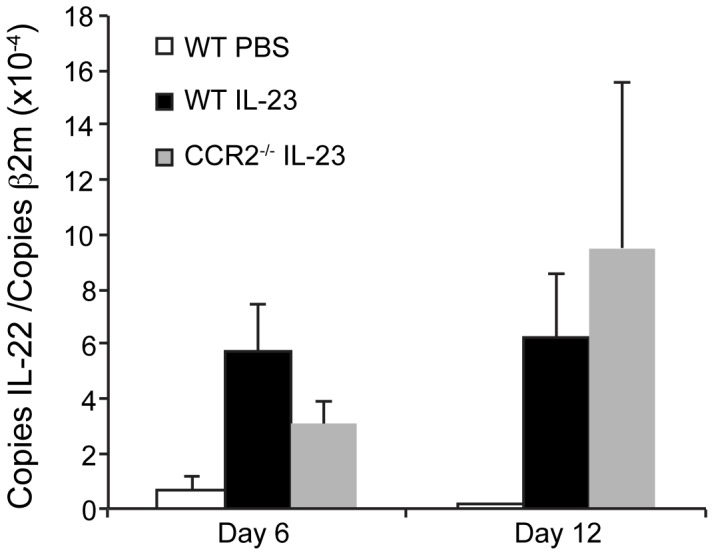
IL-22 mRNA is expressed in IL-23-injected CCR2^−/−^ mouse ears. On days 6 and 12, IL-22 mRNA was measured by real-time RT-PCR from ears of WT PBS-injected or WT and CCR2^−/−^ IL-23-injected mice. Day 6 results are an average of 11 mice per genotype in 3 experiments. Day 12 results are an average of 14 mice per genotype in 4 experiments.

Depending on the disease model, the cytokine IL-22 has been reported to have pro- or anti-inflammatory roles. Recent studies suggest that IL-22 negatively regulates allergic responses. For example, in a model of allergic airway inflammation, IL-22 decreased eosinophilia and Th2 cytokine production [47]. In contrast, in the IL-23-induced psoriasis model, IL-22 is required for the development of dermal inflammation and acanthosis [8]. In this model, psoriatic skin pathology requires early T cell-independent production of IL-22 for the subsequent, sustained T cell-dependent inflammation [9]. CCR2 is expressed on innate cells as well as activated Th17 cells, and so we examined whether CCR2 deficiency reduced IL-22 production in IL-23-injected ears. However, real-time RT-PCR analysis did not reveal any significant difference in the expression of IL-22 in the ears of WT and CCR2^−/−^ mice either early (day 6) or late (day 12) following the initiation of intradermal IL-23 injections (Figure 5). Recent studies demonstrate that IL-22-producing T cells are present in both psoriatic and chronic atopic dermatitis lesions [48], [49]. IL-22 induces keratinocyte proliferation and epidermal hyperplasia, and so may contribute to the epidermal thickening that we observe in both IL-23-injected WT and CCR2^−/−^ skin.

### Cutaneous Expression of TSLP and IL-4 is Increased in CCR2^−/−^ Mice

**Figure 6:**
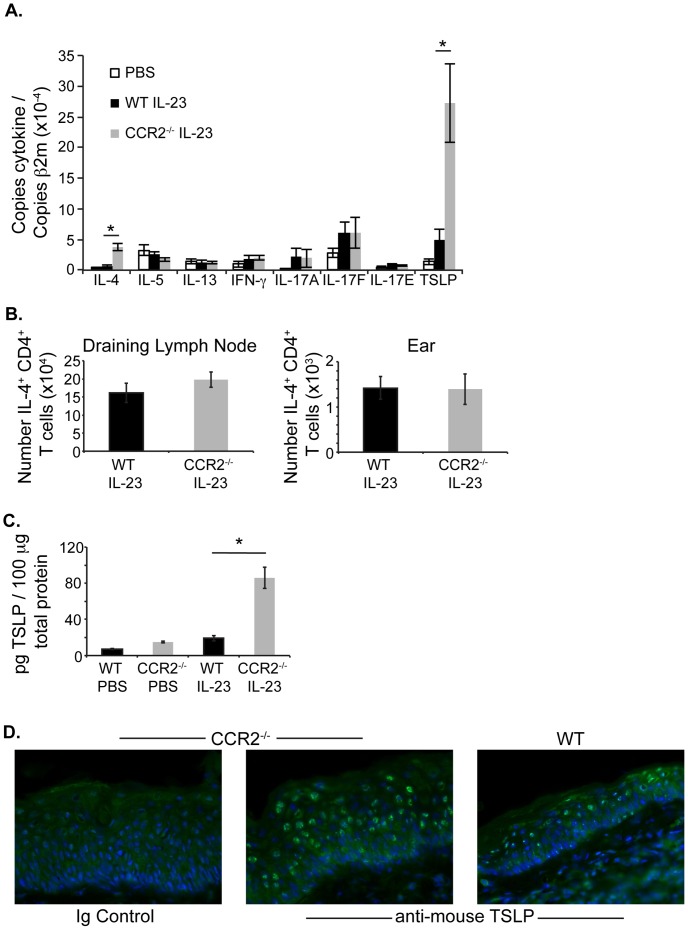
Increased TSLP and IL-4 in ears of IL-23-injected CCR2^−/−^ compared to WT mice. On day 12, (**A**) mRNA of indicated cytokines was measured by real-time RT-PCR from ears of WT PBS-injected or WT and CCR2^−/−^ IL-23-injected mice. Average of 11 mice per genotype in 3 experiments. *p<0.01. (**B**) Intracellular cytokine staining for IL-4 on gated CD3^+^ CD4^+^ T cells following stimulation with PMA and ionomycin was performed and day 12 and analyzed by flow cytometry. Number of IL-4^+^ CD3^+^ CD4^+^ T cells in draining lymph node (left panel) or IL-23-injected ear (right panel). (**C**) TSLP was measured by tissue ELISA of ears isolated from PBS-injected or IL-23-injected WT and CCR2^−/−^ mice. Average of 3 mice per genotype. *p<0.005 CCR2 KO IL-23 vs. all other groups. (**D**) Representative immunofluorescence staining of ear skin isolated on day 12 from IL-23-injected WT C57Bl/6 or CCR2^−/−^ mice. Sections were stained with Ig isotype control antibody or anti-TSLP (x600, original magnification). Data are reflective of 3 mice per genotype.

Previous studies of inflammatory responses in CCR2^−/−^ mice have observed increased Th2 cytokine production, with a corresponding decrease in Th1 cytokine expression. Although we did not detect a significant difference in the expression of IL-22 in the IL-23-injected ears of WT and CCR2^−/−^ mice, we reasoned that a decrease in other Th17 or in Th1 cytokines, or an increase in Th2 cytokines might explain the increased ear swelling and eosinophilia observed in CCR2^−/−^ IL-23-injected mouse skin. WT and CCR2^−/−^ mice were injected intradermally in the ear every other day with IL-23, and on day 12, the injected ear skin was isolated and analyzed for mRNA expression of Th1 (IFN-γ), Th2 (IL-4, IL-5, IL-13, TSLP) and Th17 (IL-17a, IL-17f) cytokines. In this model of cutaneous inflammation, we did not detect any difference in the expression of IFN-γ in the ears of IL-23-injected WT and CCR2^−/−^ mice. Similarly, expression of IL-17A and IL-17F were comparable in mice of both genotypes (Figure 6a). The IL-17 family member, IL-17E is known to induce Th2 cytokine responses [50], [51], but we failed to detect its expression in either WT or CCR2^−/−^ mice (Figure 6a). However, quantitative RT-PCR analysis demonstrated that ears of CCR2^−/−^ mice expressed increased IL-4 mRNA compared to ears of WT mice (Figure 6a). Of note, although we detected increased IL-4 mRNA expression in IL-23-injected CCR2^−/−^ ears, WT and CCR2^−/−^ draining lymph nodes and ears contained comparable numbers of IL-4-secreting T cells (Figure 6b). However, we did measure increased mRNA expression of TSLP – a cytokine known to promote and amplify Th2-type immune responses [52], [53], in the ears of CCR2^−/−^ mice (Figure 6a). Additionally, tissue lysate ELISA demonstrated increased TSLP protein expression in the ears of CCR2^−/−^ mice compared to WT mice (Figure 6c), providing a possible mechanistic link to the increased Th2-type immune response observed in these mice. Immunofluorescence staining confirmed TSLP expression by epidermal cells within IL-23-injected ears of both WT and CCR2^−/−^ mice (Figure 6d). We did not detect any specific TSLP staining in the dermis. Given the epidermal hyperplasia observed in CCR2^−/−^ mice, the increased TSLP detected in IL-23-injected CCR2^−/−^ mouse skin is likely produced by keratinocytes, although other cell types may also contribute to its production.

## Discussion

In this study, we examined the role of the chemokine receptor CCR2 in a murine model of IL-23-induced psoriasis. The CCR2 ligand CCL2 is expressed by keratinocytes in psoriatic plaques [26], [27], suggesting a potential role for CCR2 in psoriasis pathogenesis. In WT mice, intradermal IL-23 injection produces IL-22-dependent psoriasiform pathology, including acanthosis and dermal inflammatory infiltrates [8]. Here, we find that although IL-22 mRNA expression was comparable in the ears of IL-23-injected CCR2^−/−^ mice and WT mice, ears of CCR2^−/−^ mice eventually became more inflamed with increased ear swelling and epidermal thickening. This increased inflammation correlated with increased cutaneous TSLP and IL-4 expression and increased numbers of eosinophils within the skin of CCR2^−/−^ mice compared to WT mice.

Previous studies have also detected increased Th2-type immune responses in CCR2^−/−^ mice compared to WT mice using models of infection [34], inflammation [28], [35] and transplant [36]. The increased Th2 responses observed in CCR2^−/−^ mice have been correlated with decreased Th1-type responses. Since Th1-type and Th2-type cytokines are counter-regulatory [54], it has been speculated that the decreased Th1 response seen in CCR2^−/−^ mice leads to an increased Th2 response in these mice [55], [56]. However, in the IL-23 model of cutaneous inflammation we found comparable levels of Th1 (IFN-γ) and Th17 (IL-17, IL-22) cytokines in the inflamed skin of WT and CCR2^−/−^ mice. Rather, the increased Th2-type response we observed in CCR2^−/−^ mice correlated with increased cutaneous TSLP expression.

TSLP is an IL-7-like cytokine produced by several cell types, including keratinocytes [52], dendritic cells [57], [58] and basophils [59]. TSLP promotes Th2-type immune responses through stimulation of dendritic cells [52], [53], as well as by direct stimulation of CD4^+^ T cell [60] and mast cell [61] Th2 cytokine production. Additionally, TSLP promotes eosinophil survival and cytokine secretion [62]. Mice injected intradermally with TSLP [63] or transgenic for keratinocyte-specific TSLP expression [64] display hallmark features of atopic dermatitis, including Th2 cytokine production, localized edema, acanthosis, hyperkeratosis and a dermal mononuclear cell infiltrate rich in eosinophils and mast cells. In this study, we found that TSLP was increased in the skin of CCR2^−/−^ mice compared to WT mice. Similar to mice injected intradermally with TSLP or genetically engineered to overexpress TSLP in keratinocytes, the increased TSLP expression seen in CCR2^−/−^ mice correlated with development of an atopic dermatitis-like cutaneous inflammation. We found that a Th2-type immune response, including increased IL-4 expression and accumulation of eosinophils and mast cells developed in IL-23-injected CCR2^−/−^ mouse skin, likely as a result of increased TSLP expression, but independent of an increased accumulation of Th2 cells. Similarly, mice overexpressing TSLP but lacking T cells also develop atopic dermatitis [64].

While TSLP instructs dendritic cells to promote the differentiation of naïve T cells into Th2 effector cells, we did not observe an increase in Th2 cell numbers in IL-23 injected skin or draining lymph nodes of CCR2^−/−^ mice. However, TSLP also acts on innate immune cells, including mast cells and eosinophils to promote Th2-type immune responses [61], [62]. We do not yet know whether TSLP acts upstream to direct, or downstream to amplify the Th2 response in CCR2^−/−^ mice. CCR2 deficiency may change the composition of cells within the inflamed skin, and thereby the cytokines that regulate TSLP expression. In our studies, IL-23 may induce the recruitment of IL-4-producing eosinophils into the skin through the regulation of CCL7 [65], a CCR3 ligand [44], [45]. CCR3 remains intact in CCR2^−/−^ mice and is expressed on basophils, mast cells and eosinophils [46], providing a potential mechanism for the recruitment of eosinophils and mast cells into CCR2^−/−^ skin. Indeed, we detected increased CCL7 expression in CCR2^−/−^ mice compared to WT mice. In parallel, IL-23 may promote the development of Th17 cells, which induce IL-1 and TNFα production. Synergy of IL-4 with these inflammatory cytokines could induce TSLP production by keratinocytes [66], resulting in the amplification of the cutaneous Th2-type immune response. To our knowledge, this is the first study to correlate the increased Th2-type immune response in CCR2^−/−^ mice with increased TSLP production.

In conclusion, CCR2 deficiency exacerbates cutaneous inflammation induced by intradermal IL-23 injection. Although CCR2 blockade has ameliorated several inflammatory diseases by skewing T cell responses to Th2, intradermal injection of CCR2^−/−^ mice results in an atopic dermatitis-like lesion rather than a psoriasis-like lesion. CCR2-deficiency leads to more severe cutaneous inflammation with increased TSLP and Th2-type cytokine production as well as eosinophil and mast cell infiltration. These results suggest that increased TSLP expression in CCR2^−/−^ mice may contribute to the propensity of these mice to develop exaggerated Th2-type immune responses.
